# The relationships among self-care, dispositional mindfulness, and psychological distress in medical students

**DOI:** 10.3402/meo.v20.27924

**Published:** 2015-06-24

**Authors:** Jessica Slonim, Mandy Kienhuis, Mirella Di Benedetto, John Reece

**Affiliations:** 1 Faculty of Education, Monash University, Melbourne, Australia; 2 School of Health Sciences, RMIT University, Melbourne, Australia; 3 School of Psychological Science, Australian College of Applied Psychology, Melbourne, Australia

**Keywords:** dispositional mindfulness, self-care, psychological distress, medical students, intervention

## Abstract

**Background:**

Past research suggests that medical students experience high levels of psychological distress.

**Objective:**

The aim of the current study was to investigate the relationships among engagement in self-care behaviours, dispositional mindfulness, and psychological distress.

**Methods:**

The sample consisted of 139 female and 68 male Australian medical students (*N*=207) aged 17–41 years (*M*=21.82, *SD*=3.62) across the 5 years of the Monash University medical course. Participants completed an online survey comprising a demographics questionnaire, the Five Facet Mindfulness Questionnaire, the Health-Promoting Lifestyle Profile II, and the Depression, Anxiety, and Stress Scales.

**Results:**

Results revealed significant and interpretable multivariate correlations between distress and both mindfulness and self-care. Furthermore, the dispositional mindfulness observation subscale was found to be a significant moderator of the relationship between several dimensions of self-care and psychological distress.

**Conclusions:**

The present study points to the potential of self-care and mindfulness to decrease medical student distress and enhance well-being.

High levels of stressors experienced by medical students are associated with a higher incidence of psychological distress in this group (1). Distress can have a profound impact on students’ personal and professional lives. For example, distress may contribute to poor academic performance, increased drop-out rates from medical school, broken relationships, substance abuse, and suicide (2). Distress may also contribute to cynicism, reduced quality of patient care, burnout, and ultimately, a change in the culture of the medical profession (2, 3). Mindfulness and self-care are two personal factors identified in the literature as predictors of distress (4–6) that could also be targeted in future interventions.

Medical students experience a higher incidence of psychological distress throughout their medical training and as they enter the workplace (2, 3, 7). Compared with their age-matched student peers, medical students have higher rates of depression symptomatology: up to one-quarter exhibit substantial depressive symptoms (7, 8). They also display substantially higher stress levels than age-matched non-medical students (8, 9).

Some studies report more distress among female medical students than males (10, 11), while others have observed higher levels of distress among males compared with females on some measures (12). Some studies, however, have observed no gender differences in medical student distress (8).

Further research is needed to investigate the psychological mechanisms that moderate the relationships between student stressors and distress. Identification of these psychological mechanisms is likely to highlight strategies to assist medical students to cope with stressors and thereby reduce distress outcomes. There is growing interest in the role of mindfulness and self-care as coping strategies to enhance well-being and reduce medical student distress.

Despite extensive references to mindfulness in the literature, there remains disagreement over how to operationally define this concept. Mindfulness has been defined as a cognitive style that facilitates non-judgemental awareness (13). While regular practice induces mindfulness as a temporary state (14), even without practice, individuals have the capacity to be mindful and thus it can also be considered a trait. The conceptualisation of mindfulness as a dispositional trait refers to a natural ability of consciousness and self-awareness that occurs independently of training (15). This capacity may be more strongly present in some individuals than others (16).

The benefits associated with increased mindfulness through training have been the focus of considerable research in recent years (16–19). There has been less research, however, on the benefits of dispositional mindfulness. Some studies demonstrate that the well-being outcomes associated with mindfulness practice are also associated with the higher levels of dispositional mindfulness that can be present in individuals who have no prior training in mindfulness practice (15, 16, 20).

In a series of three studies stemming from their development of the Mindfulness Attention Awareness Scale, a scale that assesses individual differences in the frequency of mindfulness over time, Brown and Ryan (16) observed that individuals with high levels of dispositional mindfulness displayed greater emotional well-being. Their results also provide support for the self-regulatory capacity of mindfulness. That is, they observed that individuals with high levels of mindfulness reported greater autonomy in their daily activities and greater concordance between implicit and explicit affective states.

While there is no research to the authors’ knowledge examining the benefits of dispositional mindfulness for medical students, there is research that links mindfulness-based interventions to reduced distress in medical students. Shapiro et al. (6) evaluated an 8-week Mindfulness-Based Stress Reduction (MBSR; 21, 22) programme offered to medical and premedical students. MBSR includes training in techniques designed to increase state mindfulness and has demonstrated efficacy with other populations (20). The results of the randomised controlled trial with medical students revealed significant decreases in depression and anxiety, and significant increases in empathy and spiritual experiences at the end of the 8-week intervention. However, there was no follow-up on the long-term effects of the intervention.

Additional to the stressors inherent in medical training are the greater demands and new structure to daily life that comes with the autonomy of university life and living independently (23). The coping behaviours students develop at this stage shape their lifestyle pattern into adulthood (24). Self-care is conceptualised as health-promoting behaviours to maintain or improve well-being (25). These self-initiated behaviours include seeking health care as appropriate, adhering to a healthy diet, engaging in physical activity, participating in spiritual or religious practice, engaging with social relationships, and seeking guidance or counselling when necessary (26).

The relationship between self-care behaviours and well-being outcomes is well established (5, 24, 27). Self-care behaviours help in reducing the build-up and effects of stress and improve one's general well-being (5). Health professionals risk distress, burnout, and suboptimal professional performance if sustained stressors are not alleviated by self-care practices (28).

The research reviewed suggests that mindfulness and self-care can positively impact well-being; however, less is known about the relationship between these factors, and whether they may have an interactive effect on psychological distress. Although some research has considered the mediating role of mindfulness in the relationship between self-care and distress (5), there still remains the clinically and theoretically relevant question of whether there is a moderating relationship between mindfulness and self-care on distress, which does not appear to have been investigated. As coping strategies, both mindfulness and self-care could conceivably play a ‘buffering’ role that modulates the impact of distress, rather than mediating it. Furthermore, if it is accepted that both mindfulness and self-care have the potential to directly and independently impact distress in medical students, and if it is accepted that these two constructs share some overlapping features, then a plausible question is raised regarding the potential interactive effect of mindfulness and self-care on distress, which could be addressed by a moderation analysis.

Hence, the first aim of the current study was to examine in detail the pattern of relationships among engagement in self-care behaviours, dispositional mindfulness, and distress in Australian medical students. In an extension of previous work in this area, canonical correlation was used to explore the pattern of multivariate relationships among these variables. It was hypothesised that, at both the univariate and multivariate levels, higher levels of dispositional mindfulness would be associated with lower levels of distress, and that higher levels of self-care would be associated with lower levels of distress, although the exact pattern of these relationships could not be specified.

The second aim of the current study was to explore the potential moderating role of mindfulness on the relationship between self-care and distress. The lack of an extensive research base informing this question makes it difficult to justify specific hypotheses, but we were confident that there is theoretical support for the hypothesis that mindfulness moderates the relationship between self-care and distress. Figure 1 illustrates the nature of the moderating relationship.

**Fig. 1:**
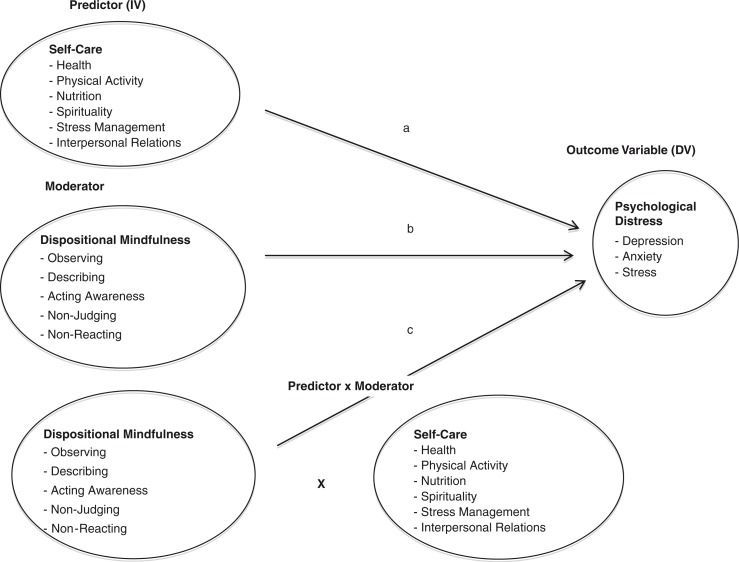
Dispositional mindfulness as a moderator of the relationship between self-care and distress.

A third aim was to consider whether there were any gender differences in self-care behaviours, dispositional mindfulness levels, or distress levels in medical students.

## Method

### Participants

The sample consisted of undergraduate medical students from Monash University, Melbourne, Australia. Participants were recruited from all 5 years of the medical degree, using an advertisement sent to students’ university email addresses. Of the approximately 1,500 students who were invited to participate in the study, 276 students commenced the survey and 207 completed the survey (13.8% response rate). The final sample comprised 139 females (67.1%) and 68 males (32.9%) aged between 17 and 41 years (*M*=21.82, *SD*=3.62).

### Materials

#### Demographic questionnaire

Participants provided demographic information including gender, age, cultural background, year in medical degree, and study setting (university or clinical).

#### Self-care

Self-care frequency was measured using the Health-Promoting Lifestyle Profile II (HPLP; 26). The HPLP II is a 52-item inventory measuring six sub-dimensions of health-promoting lifestyle: health responsibility, nutrition, physical activity, spiritual growth, interpersonal relationships, and stress management. Responses are given on a four-point scale ranging from 1 (*never*) to 4 (*routinely*). The scale developers revised the original HPLP to improve content validity. Factor analysis was used to measure construct validity and confirmed the six-dimensional structure. Criterion validity was assessed using correlations with other measures of perceived health status and quality of life (*r*'s=0.27–0.49). The scale has adequate internal consistency across the total scale (alpha coefficient=0.94) and the subscales (alpha coefficients ranged from 0.79 to 0.87). Test–retest reliability for the total scale is 0.89 (25, 29). An overall health-promoting lifestyle score is calculated for each participant by adding scores from all 52 responses, and individual subscale scores are calculated by adding responses specific to the subscale.

#### Dispositional mindfulness

The Five Facet Mindfulness Questionnaire (FFMQ; 30) was used to measure dispositional mindfulness. This scale consists of 39 items scored on a five-point Likert scale, ranging from 1 (*never/very rarely true*) to 5 (*very often/always true*). It is based on a factor analysis of five separately developed mindfulness scales, which identified five factors representing elements of mindfulness: observing (e.g., ‘I notice the smells and aromas of things’), describing (e.g., ‘I have trouble thinking of the right words to express how I feel about things’), acting with awareness (e.g., ‘When I do things, my mind wanders off and I'm easily distracted’), non-judging of inner experience (e.g., ‘I believe some of my thoughts are abnormal or bad and I shouldn't think that way’), and non-reactivity to inner experience (e.g., ‘I watch my feelings without getting lost in them’). These five scales display moderate to good internal consistency, with alpha coefficients ranging from 0.75 to 0.91 (30). Composite and individual subscale scores are calculated for participants by adding scores from all the responses and from each subscale, respectively.

#### Distress

Distress was assessed using the Depression, Anxiety, and Stress Scales (DASS; 31); a set of three self-report scales (depression, anxiety, and stress) containing 14 items scored on a four-point frequency/severity scale ranging from 0 (*did not apply to me*) to 3 (*applied to me very much, or most of the time*). Each scale is divided into subscales of 2–5 items: the Depression scale assesses dysphoria, hopelessness, devaluation of life, self-deprecation, lack of interest/involvement, anhedonia, and inertia; the Anxiety scale assesses autonomic arousal, skeletal muscle effects, situational anxiety, and subjective experience of anxious affect; the Stress scale assesses the levels of chronic non-specific arousal – difficulty relaxing, nervous arousal, being easily upset/agitated, irritability/over-reactivity, and impatience. The total scale has good internal consistency, with an alpha coefficient score of 0.97 (32). Alpha coefficients for the Depression, Anxiety, and Stress subscales are 0.94, 0.88, and 0.93, respectively (33). The subscales demonstrate convergent validity with other measures of depression (32) and anxiety (34). For the current study, composite and individual scale scores were calculated for each participant.

### Procedure

Ethical approval was obtained from both the RMIT University and Monash University Human Research Ethics Committees, and permission to recruit medical students was obtained from the Monash Medical Faculty. Students were then invited to participate in the study via an email sent to all Monash medical students at the beginning of the second semester.

Respondents were provided with the link to a website that included an online survey using Qualtrics software. Participants were first instructed to read a plain language statement, outlining the purpose of the study. Submission of the online survey implied consent. Participants then completed the demographics survey, FFMQ, HPLP II, and DASS, which were randomised to minimise order effects. The questionnaires took approximately 20 min to complete.

### Data analysis and sample size considerations

Canonical correlation was chosen to investigate the pattern of relationships among self-care, dispositional mindfulness, and distress. Canonical correlation is essentially a multivariate extension of Pearson's bivariate correlation, where the relationship between two sets of variables is examined (as opposed to two individual variables). The key findings to emerge from this analysis are, first, whether there is an overall significant multivariate relationship between the two sets of variables (e.g., between mindfulness and distress) and, second, what particular combinations of the two sets of variables are strongly related. Canonical correlation is a useful technique for an initial exploration of the pattern of relationships between two sets of variables, such as two sets of sub-tests associated with separate constructs (35).

Specific formulae for power calculations for canonical correlation are not readily available, but Tabachnick and Fidell (35) recommend a ratio of 10 participants per variable as one that provides a suitable level of power. Given our 17 variables, our final sample of 207 participants easily meets this requirement.

Canonical correlation was chosen over more sophisticated options, such as structural equation modelling (SEM), for several reasons. First, our sample was not considered large enough to conduct a reliable SEM. Second, the analysis of the relationships among these variables is in its early stages, and we did not consider our theoretical ideas regarding the overall pattern of relationships among these variables to be adequately refined for a model testing analysis, although we do speculate on a possible model linking these variables later in the article.

Our second aim, focussing on the moderating role of mindfulness in the relationship between self-care and distress, was analysed using the classic Baron and Kenny (36) approach to testing moderation, which involves running a series of models with the distress subscales forming the outcome measure, and various interactions between mindfulness and self-care subscales as the predictors. A significant interaction between the mindfulness and self-care subscale provides evidence of moderation. Moderation in this sense refers to a situation where the relationship between two variables depends on the level of a third variable, the moderator. In this instance, we want to test the hypothesis that the relationship between self-care and distress would vary according to levels of dispositional mindfulness. This can be contrasted with mediation analysis, which involves testing the hypothesis that the relationship between two variables ‘works through’ a third variable as part of a proposed theoretical causal pathway, which was not of interest to us in this study.

A power analysis indicated that, for multivariate models of this type, small-to-moderate effects would have an 80% chance of being detected with a sample of 150 participants.

Our third aim, gender differences, was analysed using single-factor between-subjects multivariate analysis of variance, with the sets of subscales for our three main measures forming the dependent outcomes. For these analyses, a power analysis indicated that small-to-moderate effects would have an 80% chance of being detected with a sample of 130 participants.

The multivariate approach to these analyses provided protection against over-inflation of the familywise error rate caused by multiple testing (35).

## Results

### Missing data, outliers, and assumption testing

Data were exported from the Qualtrics website into IBM SPSS (version 20), for analysis. Given the relatively small amount of missing data and the large sample size, missing values were not imputed, resulting in 207 complete cases for analysis. Of the 276 participants who commenced the online survey, 25% did not fully complete the FFMQ, HPLP II, or DASS. Analysis of non-completers did not find any significant differences between those who completed the survey and those who did not on any of the 17 outcome variables.

### Descriptive outcomes

Descriptive results for participants’ responses to the FFMQ subscales and HPLP II subscales are presented in Table 1. The mean scores and standard deviations for the 5-year levels and total sample responses to the DASS are presented in Table 2. All year levels were in the normal range in the severity-rating index for the DASS subscales, which are based on normative data from Lovibond and Lovibond's (31) Australian sample that predominantly comprised students (Depression: 0–9; Anxiety: 0–7; and Stress: 0–14). However, the mean scores for the depression and anxiety subscales in the present sample were higher than norms presented by Crawford and Henry (32), which were derived from a general adult population: Depression (*M=*5.55, *SD*=7.4) and Anxiety (*M=*3.56, *SD*=5.39). A single-sample *t*-test found the current sample mean for anxiety to be significantly higher than the norm value reported by Crawford and Henry, *t*(206)=3.84, *p*<0.001; the corresponding comparison for depression was not significant.

**Table 1:** Means and standard deviations for HPLP II, FFMQ, and subscales for total sample

	Total (*N*=207)	
	
Variable	*M*	*SD*
Total mindfulness	123.97	20.58
Observing	23.00	6.44
Describing	28.00	6.27
Acting with awareness	26.20	6.42
Non-judging	26.57	8.08
Non-reactivity	20.19	4.80
Total self-care	135.35	20.69
Health	18.85	4.90
Physical activity	19.76	5.44
Nutrition	24.43	4.96
Spirituality	25.87	5.04
Interpersonal relations	27.68	5.15
Stress management	18.76	3.78

*Note*. Males, *n=*68; females, *n=*139. Total Mindfulness is the Five Facet Mindfulness Questionnaire (FFMQ); Observing, Describing, Acting with Awareness, Non-judging, Non-reactivity are subscales. Total Self-care is the Health-Promoting Lifestyle Profile II (HPLP II); Health, Physical Activity, Nutrition, Spirituality, Interpersonal Relations, and Stress Management are subscales.

**Table 2:** Means and standard deviations for DASS scales for year levels and total sample

	Total (*N=*207)		First year (*n=*46)		Second year (*n=*41)		Third year (*n=*34)		Fourth year (*n=*48)		Fifth year (*n=*38)	
	
Variable	*M*	*SD*	*M*	*SD*	*M*	*SD*	*M*	*SD*	*M*	*SD*	*M*	*SD*
Depression	6.52	7.54	5.17	5.87	6.41	6.34	7.68	8.85	7.71	9.10	5.71	7.09
Anxiety	5.23	6.25	4.80	5.84	4.63	5.59	6.91	7.78	5.81	6.27	4.05	5.73
Stress	8.63	7.41	8.09	7.38	8.54	6.57	8.85	7.96	9.00	7.52	8.74	7.98

*Note*. Males, *n=*68; females, *n=*139. Depression, Anxiety, and Stress are scale scores.

### First aim: the pattern of relationships among distress, self-care, and mindfulness

Table 3 presents the inter-correlations among all measures. Of note, of the 136 correlations, only 15 were non-significant. Interestingly, 12 of these involved two subscales: the Observing subscale of the FFMQ and the Health Responsibility Subscale of the HPLP. Of the significant correlations, 93 of the 136 correlations were significant at *p*<0.001. All correlations were in the expected direction, with negative relationships observed between the DASS and both the HPLP and FFMQ, and positive relationships among all FFMQ and HPLP subscales and totals.

**Table 3:** Correlation matrix for Distress, Mindfulness, and Self-care subscales

Scale	1	2	3	4	5	6	7	8	9	10	11	12	13	14	15	16	17
1. DASS Total	*–*	0.84***	0.88***	0.93***	−0.44***	0.09	−0.24**	−0.35***	−0.50***	−0.38**	−0.33***	−0.06	−0.18*	−0.17*	−0.45***	−0.24***	−0.25***
2. DASS Anxiety		*–*	0.59***	0.70***	−0.34***	0.13	−0.24**	−0.26***	−0.43***	−0.24**	−0.30***	−0.09	−0.15*	−0.17*	−0.38***	−0.23**	−0.27***
3. DASS Depression			*–*	0.72***	−0.41***	0.06	−0.18**	−0.36***	−0.47***	−0.34***	−0.34***	−0.05	−0.16*	−0.16*	−0.51***	−0.27***	−0.29***
4. DASS Stress				*–*	−0.41***	0.06	−0.21**	−0.31***	−0.46***	−0.41***	−0.26***	−0.02	−0.16*	−0.12	−0.32***	−0.15**	−0.36***
5. FFMQ Total					*–*	0.49***	0.66***	0.73***	0.67***	0.68***	0.55***	0.36***	0.32***	0.27***	0.51***	0.46***	0.44***
6. FFMQ Observing						*–*	0.30***	0.11	−0.07	0.32***	0.35***	0.46***	0.19**	0.27***	0.17*	0.13	0.25***
7. FFMQ Describing							*–*	0.37***	0.22**	0.23**	0.46***	0.37***	0.20**	0.22**	0.39***	0.51***	0.26***
8. FFMQ Awareness								*–*	0.44***	0.41***	0.38***	0.15*	0.26***	0.22**	0.39***	0.32***	0.30***
9. FFMQ Non-judging									*–*	0.39***	0.21**	<0.01	0.11	0.04	0.30***	0.26***	0.20**
10. FFMQ Non-reactivity										*–*	0.43***	0.24***	0.32***	0.17*	0.44***	0.25***	0.46***
11. HPLP Total											*–*	0.73***	0.67***	0.68***	0.78***	0.68***	0.71***
12. HPLP Health												*–*	0.38***	0.44***	0.42***	0.44***	0.43***
13. HPLP Physical													*–*	0.57***	0.31***	0.16*	0.40***
14. HPLP Nutrition														*–*	0.33***	0.19**	0.33***
15. HPLP Spirituality															*–*	0.66***	0.58***
16. HPLP Interpersonal																*–*	0.40***
17. HPLP Stress																	*–*

*Note. N=*207.

* *p*<0.05

** *p*<0.01

*** *p*<0.001

DASS, Depression, Anxiety, and Stress Scale; FFMQ, Five Facet Mindfulness Questionnaire; HPLP, Health-Promoting Lifestyle Profile II.

The results for the canonical correlation analysis between mindfulness and distress are presented in Table 4. In addition to the canonical loadings (*r*) and standardised canonical coefficients (*b*), the table also presents the canonical correlation values and a summary of the redundancy analysis for each significant canonical variate. The first canonical correlation was 0.59; the second was 0.24 (accounting for 35 and 6% of overlapping variance, respectively). The third canonical correlation was 0.16. The three canonical correlations combined accounted for 43% of variance between the two sets of variables. Both the first, Λ=0.61, χ^2^(15, *N*=207)=101.38, *p*<0.00, and the second, Λ=0.92, χ^2^(8, *N*=207)=16.41, *p*=0.04, of the three canonical correlations were significant. Hence, the first two sets of canonical variates are reported. However, as is often the case with canonical correlation, the proportion of variance, redundancy, and significance test results clearly indicate that the first canonical variate contributed most to the overall relationship between the two sets of variables (35); hence, interpretation focussed on the first canonical variate pair.

**Table 4:** Summary of canonical correlation between psychological distress variables and dispositional mindfulness variables

	First canonical variate		Second canonical variate	
	
	*r*	*b*	*r*	*b*
Psychological distress set				
Depression	0.90	0.43	−0.01	0.13
Anxiety	0.81	0.24	0.51	1.33
Stress	0.93	0.46	−0.25	−1.28
Proportion of variance	0.78		0.11	Total=0.89
Redundancy	0.27		0.01	Total=0.28
Dispositional mindfulness set				
Observing	0.14	0.33	0.44	0.28
Describing	−0.39	−0.21	−0.33	−0.52
Acting awareness	−0.60	−0.13	−0.01	−0.06
Non-judging	−0.87	−0.56	−0.19	−0.38
Non-reacting	−0.67	−0.45	0.70	0.90
Proportion of variance	0.35		0.17	Total=0.52
Redundancy	0.12		0.01	Total=0.13
Canonical correlation	0.59		0.24	

For the first canonical variate, the results effectively mirrored what was found with the bivariate correlations: low values on depression, anxiety, and stress were associated with higher self-reported scores on all of the dispositional mindfulness subscales, except for observation, which, with a value of 0.14, was under the criterion of 0.30 for noteworthiness (35). The strongest unique relationship was evident between the distress subscales and the mindfulness non-judgemental subscale.

Because of its low values for redundancy and proportion of variance explained, the second canonical variate was not interpreted.

The results for the canonical correlation analysis for distress and self-care are shown in Table 5. The first canonical correlation was 0.57; the second was 0.35 (accounting for 32 and 12% of overlapping variance, respectively). The third canonical correlation was 0.09. The three canonical correlations combined accounted for 45% of variance between the two sets of variables. Both the first, Λ=0.59, χ^2^(18, *N*=207)=105.99, *p*< 0.001, and the second, Λ=0.87, χ^2^(10, *N*=207)=27.30, *p*=0.002, of the three canonical correlations were significant. Hence, the first two sets of canonical variates are reported. Unlike what was found with the first canonical correlation, there was evidence from the significance levels, proportion of variance explained, and redundancy figures to support interpretation of both canonical variate pairs.

**Table 5:** Summary of canonical correlation between psychological distress variables and self-care variables

	First canonical variate		Second canonical variate	
	
	*r*	*b*	*r*	*b*
Psychological distress set				
Depression	0.95	1.05	0.24	−0.64
Anxiety	0.68	0.37	0.28	−0.45
Stress	0.58	−0.44	0.81	1.59
Proportion of variance	0.57		0.26	Total=0.83
Redundancy	0.18		0.03	Total=0.21
Self-care set				
Health responsibility	−0.14	0.34	0.12	0.38
Physical activity	−0.27	−0.02	−0.24	−0.11
Nutrition	−0.32	−0.12	−0.02	0.12
Spiritual growth	−0.95	−1.13	−0.03	0.39
Relationships	−0.54	0.05	0.12	0.17
Stress management	−0.44	0.11	−0.76	−1.21
Proportion of variance	0.26		0.11	Total=0.37
Redundancy	0.09		0.01	Total=0.10
Canonical correlation	0.57		0.35	

For the first canonical variate, low values on depression, anxiety, and stress were strongly associated with higher self-reported scores on stress management, relationships, and particularly spiritual growth, which exhibited the largest correlation and coefficient values. The relationship between distress and both nutrition and physical activity was moderate, with a weak relationship evident between distress and health responsibility. Overall, psychological and psychosocial self-care variables were more strongly related to distress than physical self-care measures.

The second canonical variate revealed a simple relationship between stress and stress management in the expected negative direction. It is interesting to note that this relationship was strong enough to be revealed as a stand-alone meaningful canonical variate pair.

### Second aim: dispositional mindfulness as a moderator of the relationship between self-care and distress

The moderating effect of dispositional mindfulness on the relationship between self-care and distress was evaluated by running a series of multivariate regression models. For each model, the dependent measure was made up of the three distress subscales. Models involving every combination of the self-care and mindfulness subscales were tested, 30 models in all. In each case, the model consisted of a self-care subscale, a mindfulness subscale, and the interaction between the two, which was the test of moderation. A general linear modelling approach (36) was taken over a bootstrapping approach (37), because of the perceived importance of the multivariate outcome, which currently is not available using bootstrapping.

Of the 30 models tested, five significant multivariate interactions were observed, and these are reported in Table 6, along with the associated univariate outcomes for each distress subscale. Of note, three of the five significant outcomes revealed mindfulness observation to be a significant moderator. This is particularly interesting, given that observation was not found to have a significant bivariate relationship with distress. Also of note was the finding that spiritual growth was moderated by three of the mindfulness subscales; recall that spiritual growth was the self-care subscale that revealed the strongest relationship with distress.

**Table 6:** Summary of multivariate and univariate moderation analysis showing results for significant multivariate outcomes only

			Multivariate result				Univariate result		
			
Moderator	Predictor	Distress subscale	Λ	*F*	*p*	$\eta_{\text{p}}^{2}$	*F*	*p*	$\eta_{\text{p}}^{2}$
Observing	Relationships		0.95	3.28	0.02	0.05			
		Depression					0.18	0.67	<0.01
		Anxiety					3.31	0.07	0.02
		Stress					2.78	0.10	0.01
Observing	Health responsibility		0.96	2.89	0.04	0.04			
		Depression					0.48	0.49	<0.01
		Anxiety					3.58	0.06	0.02
		Stress					0.11	0.74	<0.01
Observing	Spiritual growth		0.94	4.09	0.008	0.06			
		Depression					2.29	0.13	0.01
		Anxiety					7.36	0.007	0.04
		Stress					10.74	0.001	0.05
Describing	Spiritual growth		0.95	3.47	0.02	0.05			
		Depression					0.05	0.82	<0.01
		Anxiety					0.25	0.62	<0.01
		Stress					3.95	0.02	0.02
Non-reactivity	Spiritual growth		0.95	3.40	0.02	0.05			
		Depression					3.08	0.08	0.02
		Anxiety					0.07	0.79	<0.01
		Stress					0.53	0.47	<0.01

*Note*. *df*=(3, 201) for all multivariate tests and (1, 203) for all univariate tests.

**Fig. 2:**
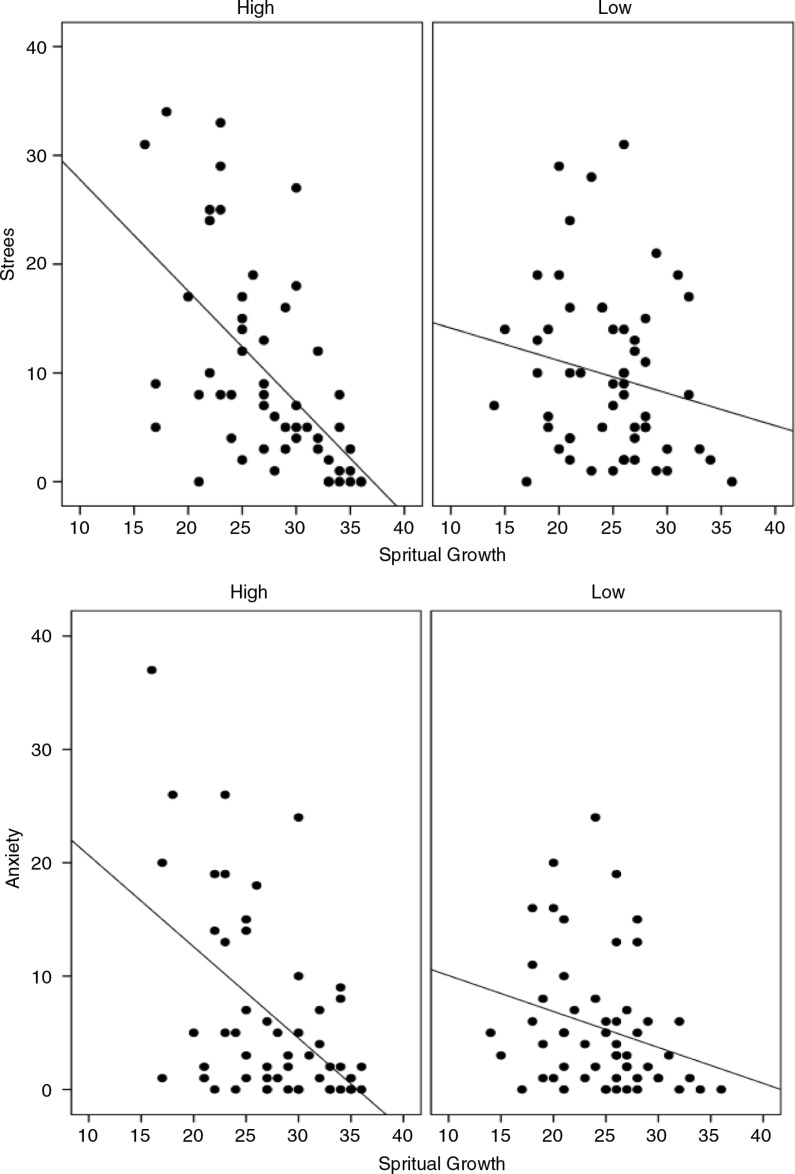
Moderating effect of dispositional mindfulness observing subscale on the relationship between spiritual growth, and anxiety and stress.

To illustrate the nature of the moderating relationship, Fig. 2 shows the relationship between spiritual growth and both anxiety and stress for two levels of observation. This relationship was chosen for illustration because (a) it revealed the strongest univariate results and (b) it involved the two most interesting variables in this analysis – observation and spiritual growth.

To create these graphs, scores on observation were sorted and grouped according to quartile splits – high and low. What is evident from these figures is that, for both anxiety and stress, the relationships with spiritual growth are much stronger for those participants who reported high levels of observation versus those who reported low levels of observation. This is confirmed by the respective *R* ^2^ values: anxiety, low observation=0.07, high observation=0.26; stress, low observation=0.04, high observation=0.33. This pattern was similar, although not as strong, for the other significant moderation results.

### Third aim: gender differences

A series of three single-factor between-subjects MANOVAs was conducted to compare distress, mindfulness, and self-care scores for males and females. For each analysis, the subscales of the relevant measure were entered as the dependent variable combination. The univariate total scores for each measure were analysed separately. No significant multivariate or univariate differences were found for distress. A significant multivariate difference was found for dispositional mindfulness, Λ=0.94, *F*(5, 201)=2.80, *p=*0.02. $\eta_{\text{p}}^{2}$=0.07, with subsequent univariate ANOVAs revealing significantly higher scores for males on the awareness and non-reactivity subscales, along with the univariate total score. Details of these univariate results are shown in Table 7. A significant multivariate effect was also found for self-care, Λ=0.90, *F*(6, 200)=3.61, *p=*0.002. $\eta_{\text{p}}^{2}$=0.10. Despite the significant multivariate result, none of the univariate tests revealed a significant gender difference.

**Table 7:** Means, standard deviations and univariate inferential test results for mindfulness, self-care, and distress for males and females

	Sex				
			
Scale	Male	Female	*F*	*p*	$\eta_{\text{p}}^{2}$
Distress					
Total	22.35 (21.86)	21.01 (18.61)	0.21	0.65	<0.01
Anxiety	5.65 (6.59)	5.02 (6.09)	0.46	0.50	<0.01
Depression	7.10 (8.73)	6.23 (6.91)	0.61	0.44	<0.01
Stress	8.38 (7.87)	8.76 (7.19)	0.12	0.73	<0.01
Mindfulness					
Total	129.85 (19.00)	121.09 (20.77)	8.59	0.004	0.04
Observing	23.90 (6.26)	22.57 (6.50)	1.95	0.16	<0.01
Describing	28.25 (6.30)	27.87 (6.28)	0.17	0.68	<0.01
Acting with awareness	27.88 (6.10)	25.38 (6.44)	7.13	0.008	0.03
Non-judging	28.15 (7.29)	25.81 (8.36)	3.89	0.05	0.02
Non-reactivity	21.68 (4.81)	19.46 (4.64)	10.17	0.002	0.05
Self-care					
Total	131.74 (21.62)	135.65 (20.29)	0.09	0.77	<0.01
Health	18.71 (4.94)	18.91 (4.90)	0.08	0.78	<0.01
Physical activity	20.38 (5.63)	19.46 (5.33)	1.31	0.25	<0.01
Nutrition	23.60 (5.01)	24.84 (4.91)	2.87	0.09	0.01
Spirituality	25.93 (5.34)	25.84 (4.91)	0.01	0.91	<0.01
Interpersonal relations	26.72 (5.38)	28.15 (4.99)	3.56	0.06	0.02
Stress management	19.40 (4.02)	18.45 (3.63)	2.92	0.09	0.01

*Note*. *N*=207 and *df*=(1, 205) for all tests.

## Discussion

The findings supported the hypothesis that higher levels of dispositional mindfulness would be associated with lower levels of distress, and that higher levels of self-care would be associated with lower levels of distress. Dispositional mindfulness and levels of distress will be discussed first. Medical student distress levels in the current study were within the normal ranges of the normative data collected from an Australian student sample (31); however, their depression and anxiety levels were higher when compared with a general adult population (32). This finding is consistent with other studies that have found higher levels of distress in medical student populations compared to the general population (2, 7).

Higher levels of dispositional mindfulness, in particular, the non-judgemental subscale, was most strongly associated with lower levels of distress. This finding is consistent with past research that found that dispositional mindfulness was negatively related to distress symptomatology (16, 30). While this study was the first to assess mindfulness as a dispositional construct in a medical student sample, the findings are consistent with Shapiro et al.'s (6) study that found significant decreases in distress symptomatology for those medical and premedical students who participated in a mindfulness programme.

The results of this study support the proposition that dispositional mindfulness has psychological benefits. Whether mindfulness is a trait (as conceptualised in the current study), or a state induced through training and practice (5, 14), positive well-being outcomes are thought to come via the same underlying processes. That is, increased moment-to-moment awareness and heightened self-regulation, which reduces patterns of automatic, mindless, and judgemental thinking, and heightened reactivity in response to situational stressors (e.g., strong negative emotional responses to thoughts and emotions; 20, 38, 39).

This study did not address the direction of causality between the variables of mindfulness and distress. The theoretical literature suggests that dispositional mindfulness can be an antecedent of distress (i.e., a protective factor against distress; 20) or consequence of distress (i.e., a coping strategy in an attempt to alleviate distress; 40). It is also possible that a bi-directional relationship exists between mindfulness and distress. Thus, further research is required to determine the mechanisms connecting the two variables and identify whether dispositional mindfulness alleviates or prevents distress, or both.

Higher levels of self-care, in particular spiritual growth, were associated with lower levels of distress. Psychological and psychosocial self-care variables were more strongly related to distress than physical self-care measures. Overall, these findings are consistent with past research that identifies the role of self-care as a moderator in the relationship between stress and well-being (5, 27).

The second hypothesis that mindfulness would moderate the relationship between self-care and distress was supported. Specifically, multivariate regression modelling revealed that the mindfulness observing subscale was a significant moderator of the relationship between three psychological and psychosocial self-care subscales – relationships, health responsibility, and spiritual growth – and distress. Furthermore, the relationship between spiritual growth and distress was moderated by observing, describing, and non-reactivity. However, the strongest relationships were among anxiety, stress, spiritual growth, and observation. There was a strong moderating effect of observing on the relationship between spiritual growth levels and both anxiety and stress levels.

Findings from the study revealed no significant gender difference in medical student distress. Past research has been inconsistent on whether females or males are more at risk of distress (10, 11, 12) . Other studies, consistent with the present study, have found no significant gender difference in medical students.

Males were found to have significantly higher mindfulness scores than females on the awareness and non-reactivity subscales. This may be explained by the fact that females ruminate on negative feelings more than males (41, 42). As mindfulness involves paying attention to both positive and negative stimuli, females may experience more difficulty being mindful than males.

The findings did not reveal any significant difference in self-care between males and females. Past research has found gender differences in self-care, although there has been inconsistency as to whether male or female university students have higher self-care levels (43, 44). Past research also highlights gender differences in particular healthy lifestyle habits among university students (e.g., a higher prevalence of unhealthy eating behaviours and attitudes among females, and a higher prevalence of alcohol consumption among males; 44, 45). Therefore, future research could look at differences between males and females in specific self-care behaviours, which may assist in fostering gender-sensitive strategies to promote a healthy lifestyle among university students.

A number of practical implications have emerged from the current study for the promotion of well-being in medical students. First, the results of the current study indicate that dispositional mindfulness and self-care behaviours are associated with lower distress in the medical student population. This highlights the importance of including mindfulness and healthy lifestyle interventions into the medical school curriculum to promote student well-being. As dispositional mindfulness can be nurtured by practice (16), it is likely that mindfulness training through mindfulness-based programmes would increase dispositional mindfulness and help reduce medical student distress. Healthy lifestyle habits can also be taught (25). Second, the results of the current study indicate that dispositional mindfulness moderates the relationship between self-care and distress. That is, the relationship between self-care and well-being is stronger in the context of greater dispositional mindfulness. Therefore, interventions aimed at reducing psychological distress should emphasise the development of mindfulness. While some individuals might become more mindful with self-care instruction, this should not be assumed. Direct instruction in mindfulness practice is likely to increase the likelihood of benefits for well-being.

Several limitations must be acknowledged in the present study. The first limitation is the potential response bias. The sample consisted of students who volunteered to participate. There may be systematic differences between those individuals who participated and those who did not, which may limit the representativeness of the sample. Distressed students may have been underrepresented in the sample, given their lower levels of motivation and increased stress and anxiety. Therefore, the sample may be skewed towards less distressed students. Overcoming this limitation in future research could be achieved by attaining a higher response rate, perhaps by having university staff promote the study, sending additional reminder emails to students, or offering incentives.

Another limitation was the self-report measures used to assess the variables. There may be discrepancies between participants’ responses and actual behaviours as a result of social desirability bias, or discomfort surrounding sensitive personal information disclosed through an online survey.

A final limitation pertains to participants’ completion of a course that included aspects of both self-care and mindfulness. The Health Enhancement Programme (HEP), an integrated mindfulness and lifestyle programme, is an incorporated part of the Monash University core-curriculum since 2002 and may have had an influence on participants’ self-care and dispositional mindfulness levels and on the patterns of relationships found in the study (46). Although the HEP was taught to all students who participated in this study, this learning occurred most recently for first-year students, as it is offered in the second half of the first semester of the medicine degree. Therefore, it is possible that first-year students in particular may have heightened self-care and mindfulness levels. The present study indicates, however, that there were no differences in distress levels across year levels. Importantly, because participants in the current study had completed the HEP, the ability to generalise the results of the current study to other medical students is limited.

It has been demonstrated that dispositional mindfulness is beneficial. The question remains: how does this cognitive process develop? There are two fundamental ideas that underlie this question: first, which individuals are more (or able to be) dispositionally mindful. The present study found significant gender differences in mindfulness levels. Future research could investigate other demographic variables that may be linked with individual differences in the development of mindfulness, for example, age, religion, or socioeconomic status. These factors may independently affect mindfulness, or perhaps act as moderators of the relationship between mindfulness and distress (47). Second, what other personal factors influence the development of mindfulness? Additional research is required to understand how psychological and lifestyle factors impact on an individual's mindfulness levels. Future research exploring the antecedents and phenomenology of mindful awareness would deepen our understanding of dispositional mindfulness (16).

The consequences of psychological distress are potentially harmful for medical students’ personal and professional lives, and may include burnout, broken relationships, drug abuse, and reduced quality of patient care. The well-being of medical students is in the interest not only of the students themselves but also of physicians, health-care organisations, and for the patients for whom they provide care. Thus, student's well-being must be a priority for medical schools, and it should be the aim of every university to create an institutional culture whereby medical students graduate as more self-aware, better skilled, and as role models of health enhancing behaviours. Future research should focus on the long-term effectiveness of mindfulness-based intervention programmes in increasing dispositional mindfulness, and assess whether dispositional mindfulness and self-care influence clinical practice.
